# Clinical Implications and Management of Spontaneous Portosystemic Shunts in Liver Cirrhosis

**DOI:** 10.3390/diagnostics14131372

**Published:** 2024-06-28

**Authors:** Simona Juncu, Horia Minea, Irina Girleanu, Laura Huiban, Cristina Muzica, Stefan Chiriac, Sergiu Timofeiov, Florin Mihai, Camelia Cojocariu, Carol Stanciu, Anca Trifan, Ana-Maria Singeap

**Affiliations:** 1 Department of Gastroenterology, Faculty of Medicine, “Grigore T. Popa” University of Medicine and Pharmacy, Universitatii Street No. 16, 700115 Iasi, Romania; simona.juncu@yahoo.com (S.J.); horia.minea@yahoo.com (H.M.); gilda_iri25@yahoo.com (I.G.); huiban.laura@yahoo.com (L.H.); lungu.christina@yahoo.com (C.M.); cameliacojocariu@yahoo.com (C.C.); stanciucarol@yahoo.com (C.S.); ancatrifan@yahoo.com (A.T.); anamaria.singeap@yahoo.com (A.-M.S.); 2 Institute of Gastroenterology and Hepatology, “St. Spiridon” Emergency County Hospital, Bd. Independentei No. 1, 700111 Iasi, Romania; 3 Department of Surgery, Faculty of Medicine, “Grigore T. Popa” University of Medicine and Pharmacy, Universitatii Street No. 16, 700115 Iasi, Romania; stimof@yahoo.com; 4 Department of Surgery, “St. Spiridon” Emergency County Hospital, Bd. Independentei No. 1, 700111 Iasi, Romania; 5 Department of Radiology and Medical Imaging, “Grigore T. Popa” University of Medicine and Pharmacy, Universitatii Street No. 16, 700115 Iasi, Romania

**Keywords:** portal hypertension, liver cirrhosis, spontaneous portosystemic shunts, variceal bleeding, hepatic encephalopathy, liver transplantation

## Abstract

Portal hypertension from chronic liver disease leads to the formation of collateral blood vessels called spontaneous portosystemic shunts (SPSS). These shunts may form from existing vessels or through neo-angiogenesis. Their location affects clinical outcomes due to varying risks and complications. This review summarizes current knowledge on SPSS, covering their clinical impact and management strategies. Recent data suggest that SPSS increases the risk of variceal bleeding, regardless of shunt size. The size of the shunt is crucial in the rising incidence of hepatic encephalopathy (HE) linked to SPSS. It also increases the risk of portopulmonary hypertension and portal vein thrombosis. Detecting and assessing SPSS rely on computed tomography (CT) and magnetic resonance imaging. CT enables precise measurements and the prediction of cirrhosis progression. Management focuses on liver disease progression and SPSS-related complications, like HE, variceal bleeding, and portopulmonary hypertension. Interventional radiology techniques such as balloon-occluded, plug-assisted, and coil-assisted retrograde transvenous obliteration play a pivotal role. Surgical options are rare but are considered when other methods fail. Liver transplantation (LT) often resolves SPSS. Intraoperative SPSS ligation is still recommended in patients at high risk for developing HE or graft hypoperfusion.

## 1. Introduction

Portal hypertension due to chronic liver disease induces a compensatory response involving the formation of collateral blood vessels known as spontaneous portosystemic shunts (SPSS) [1]. These collaterals form by reopening closed embryonic venous channels, creating a link between venous portal flow and systemic circulation, thereby bypassing the liver. An analytical model has been suggested for the compensatory mechanism and formation of SPSS. The shunt flow is defined as the ratio between portal venous pressure (PVP) and shunt resistance (SR). When PVP significantly increases due to liver cirrhosis, the shunt flow rises. Conversely, if SR decreases, shunt flow would increase, as seen in aneurysmal dilations of the vascular channels. The circuit bypass created by the shunts reduces PVP and portal blood flow [2]. Additionally, evidence suggests that SPSS are not solely derived from pre-existing vascular channels but may also result from neo-angiogenesis [3]. The formation of portosystemic collateral vessels is driven by the angiogenic process influenced by the vascular endothelial growth factor (VEGF), which can be inhibited by blocking the VEGF/VEGF receptor-2 signaling pathway. Stimuli, such as inflammation, oxidative stress, and hypoxia, promote VEGF overexpression, leading to increased angiogenesis in the splanchnic territory of portal hypertensive and cirrhotic patients. VEGF triggers nitric oxide (NO) synthesis and increases vascular permeability, which is fundamental to the initial collateralization of the portal system [4].

The prevalence of SPSS is remarkably high, as recent evidence shows. A study conducted by the Baveno VI Cooperation Group found SPSS in 60% of the investigated patients, with half of these cases involving large SPSS (greater than 8 mm) [5]. Another retrospective study reported that 63.5% of patients had SPSS, with 18% having a shunt diameter of 1 cm or more. Additionally, a retrospective cohort study revealed that the prevalence of SPSS increases with worsening liver function or portal hypertension yet remains high (46–55%) even among patients with compensated cirrhosis, preserved liver function (Model for End-Stage Liver Disease or MELD less than 10), or liver stiffness measurement less than 21 kPa. These findings highlight the significant frequency of SPSS, even in cases of compensated cirrhosis [6]. Moreover, there appears to be a connection between the etiology of liver cirrhosis and the presence of SPSS. Several studies discovered that portosystemic shunts, particularly recanalized paraumbilical vein (RPUV), were more common in patients with alcohol-associated cirrhosis, likely due to a later diagnosis in these patients [7].

Based on their location relative to the spleno-porto-mesenteric confluence, SPSS can be anatomically categorized into left-sided and right-sided (central) shunts. This anatomical classification holds specific clinical significance due to the varying risks and complications associated with each location. Left-sided SPSS include the splenorenal shunt (SRS), which is one of the most frequently discovered SPSS in patients with liver cirrhosis, as well as the gastrorenal shunt (GRS) and gastrocaval shunt (GCS). The SRS represents a direct communication between the splenic vein and the left renal vein without involving the gastrointestinal tract. Therefore, it does not contribute to the formation of varices or the risk of spontaneous bleeding. However, it can still have clinical significance by diverting blood flow away from the liver, potentially exacerbating hepatic encephalopathy. GRS is often found in patients with cardiofundic gastric varices (up to 85%) and forms a link between the gastric vein and the left renal vein (LRV), carrying a significant risk for variceal bleeding [8]. Among right-sided SPSS, the most frequent is the RPUV, which is often associated with ascites. Right-sided SPSS also include rectal varices and esophageal varices, posing substantial risks for life-threatening hemorrhage. Other, less frequent, shunts include mesocaval, mesoazygos, portocaval, portorenal, mesoiliac, and mesorenal shunts. Their association with various portal hypertensive complications has not been extensively studied, but they are likely to contribute to overall morbidity associated with advanced liver disease [9].

The objective of this review is to synthesize contemporary insights into SPSS, encompassing their clinical consequences and associated complications and offering a nuanced strategy for their effective management.

## 2. Clinical Implications

### 2.1. Variceal Bleeding

Even though there have been discussions of a potential protective role of SPSS in the development of variceal bleeding [10], recent data suggest that the presence of SPSS is not protective and is, in fact, a risk factor for variceal bleeding [11].

The presence of SPSS has a considerable impact on the blood flow patterns within the liver and splanchnic circulation. As a result of the increased portal pressure, several shunts may form to divert blood away from the liver. Although the shunts can reduce the pressure in the portal vein and in its collaterals, they can also determine changes in local hemodynamics. Anterior studies suggested that the presence of SPSS could be protective for variceal bleeding [10]. Moreover, there seems to be a relation between the size of esophageal varices and the equivalent SPSS diameter. A study that included 144 subjects with liver cirrhosis found that the participants who did not have SPSS, as well as those who had a very large SPSS diameter, had a lower occurrence of esophageal varices in comparison to patients with SPSS of a low or large diameter [12]. However, most studies concluded that the overall risk of variceal bleeding was higher in the setting of SPSS [10,11,13,14,15]. The persistence of a high risk for variceal bleeding in the setting of SPSS was attributed to the insufficient decrease in portal pressure despite the presence of shunts. This led to a similar or even elevated risk for the development of esophageal and/or gastric varices and, consequently, associated with a similar or even higher risk of variceal bleeding [13]. The seemingly contradictory findings of fewer varices but a higher risk of bleeding in patients with SPSS can be explained by the alterations in normal hemodynamics caused by SPSS. As such, the presence of SPSS was indicative of portal hypertension, with a potential decrease in portal venous flow to the liver but with no added advantage concerning the decreasing risk for bleeding [11]. All in all, the presence of SPSS could be considered an indicator of advanced portal hypertension and should consequently prioritize the management of these patients.

Concerning the hemodynamic impact of SPSS on portal hypertension, a recent retrospective study including 45 patients with gastric variceal bleeding and/or HE who received embolization found an elevation of the HVPG from 13.4 ± 3.2 at baseline to 16.9 ± 3.7 mm Hg after occlusion of the shunt (*p* < 0.001). These observations suggested that the shunt occlusion could lead to increased portal pressure; thus, patient management should be addressed by optimizing betablocker therapy [15]. Starting prophylactic betablockers in patients undergoing SPSS occlusion due to HE is a reasonable strategy. However, this decision should be individualized based on a comprehensive assessment of the patient’s clinical status and risk factors. Additionally, in patients with existing varices, the decision to occlude the shunt due to HE should be made on a case-by-case basis after a thorough assessment of the risks and benefits.

Although most patients presented a single shunt, around 20–30% of patients had multiple SPSS [5,14]. There has been a debate regarding the clinical impact of single versus multiple shunts, as well as regarding the size of the shunts. A large retrospective study including 1729 cirrhotic patients found that the presence of a large single SPSS of over 8 mm diameter was associated with more advanced liver disease as well as with more frequent complications such as HE when compared to small shunts with a diameter of under 8 mm. However, no significant difference regarding the size of the shunt was noted in terms of the rate of variceal bleeding. Patients without shunts presented fewer episodes of variceal bleeding compared to patients with SPSS [5]. A retrospective database review aimed to establish the clinical characteristics of SPSS in 222 patients with liver cirrhosis that were followed up from March 2015 to July 2019. The authors stated that although in patients with large baseline SPSS (over 1 cm) the HVPG value was lower than in participants with SPSS under 1 cm, there was no significant difference regarding previous variceal bleeding episodes. Moreover, gastric varices were predominant in patients with large SPSS. However, there was an increased risk for the development of variceal bleeding during follow-up (hazard ratio of 2.95 for a 95% confidence interval between 1.06 and 8.29, *p* = 0.039).

More recently, Praktiknjo et al. [14] assessed the influence of the diameter of individual shunts, as well as the number of shunts, on the clinical outcomes of patients with liver cirrhosis. The number of shunts was not found to be indicative of the severity of portal hypertension. Moreover, the total cross-sectional SPSS area (TSA), evaluated by radiological studies, was associated with a higher rate of poor outcomes. The authors found that a total TSA over 83 mm^2^ was associated with a higher percentage of overt hepatic encephalopathy, higher MELD scores, and lower 1-year survival. In the large TSA group, 12% of patients presented variceal bleeding compared to 19% in the small TSA group, but with no statistical significance. Thus, although the size or number of shunts may significantly impact the clinical outcome in patients with liver cirrhosis, there was not enough evidence to firmly support either the protective or deleterious role of the size or number of shunts in regard to the risk of variceal bleeding. 

In conclusion, the available data do not support the protective role of SPSS for variceal bleeding. There is increasing evidence that SPSS is associated with a higher risk of variceal bleeding, regardless of the size of the shunts.

### 2.2. Neurological Complications

Hepatic encephalopathy (HE) is a common complication among patients with cirrhosis, representing a potentially reversible neurological condition arising from hepatocellular dysfunction and portosystemic shunting, which leads to increased toxin levels [16]. The correlation between SPSS and HE is well-established and supported by numerous studies. Complementary investigations have revealed that a significant proportion (46–71%) of patients experiencing recurrent and/or persistent HE exhibit large SPSS. Furthermore, several authors have noted that SPSS are present in 71% of cases of cirrhotic patients with chronic HE unresponsive to standard medical treatment [10]. A large retrospective cohort study involving 1729 cirrhotic patients reported that episodic HE occurred in 48% of patients with large SPSS, 34% patients with small SPSS, and 20% without SPSS, while recurrent or persistent HE was observed in 52% of patients with large SPSS, 44% of patients with small SPSS, and 37% of patients without SPSS, with these data indicating a close association between the size of SPSS and the occurrence of episodic, recurrent, or persistent HE [5].

Furthermore, a multicenter retrospective study suggested that the prevalence of SPSS increases with worsening liver function. Screening for SPSS has consistently identified patients at high risk of developing serious complications necessitating intensive care unit management [17]. In cases where HE lacks precipitating factors and fails to respond to medical treatment, SPSS diagnosis should be considered, supported by recent data showing control of the HE following shunt embolization [18].

The size of the shunt appears to be an important factor that contributes to the increasing incidence of HE associated with SPSS. Patients with large SPSS experience a higher frequency of HE episodes (47% vs. 33%, *p* < 0.05) and exhibit lower one-year survival rates compared to those with small SPSS (69% vs. 84%, *p* < 0.001) [14].

Another significant factor contributing to the development of HE is the presence of hepatofugal blood flow in SPSS, which, in conjunction with the size of the shunt, facilitates the bypass mechanism. Consequently, in these patients, the onset of HE may not necessarily require a precipitating factor, as the presence of SPSS acts as a predisposing factor. Nevertheless, the development of HE cannot be solely attributed to SPSS, as it is rarely observed in patients with noncirrhotic portal hypertension [19]. 

Patients with large SPSS have been shown to develop a bradykinetic–rigidity syndrome, also named “cirrhosis-related parkinsonism”. These patients exhibit cognitive dysfunction, ataxia, dystonia, choreoathetosis, or spastic paraparesis [15]. Research conducted on cirrhotic patients has revealed clinical extrapyramidal manifestations in the majority of patients (57 out of 98, 59.4%), with bradykinesia being the most common sign. Moreover, advanced liver cirrhosis appears to be associated with parkinsonism-like symptoms. The progression of these symptoms, despite medical treatment for HE, suggests the presence of cirrhosis-related parkinsonism [20]. The therapeutic approach to this form of HE is focused on reducing systemic and central inflammation, as well as decreasing brain ammonia levels and GABAergic tone [21].

A rare complication associated with its development is hepatic myelopathy (HM). Patients with HM exhibit progressive, symmetric spastic paraparesis, myasthenia gravis, and extensive portal collateral circulation. Additionally, bilateral symmetric demyelinating lesions of the lateral cord of the spinal cord may occur, which can explain why the upper limbs of patients with HM are affected rather than the lower limbs. The HM diagnosis is primarily one of exclusion, as other neurologic pathologies must be ruled out, whereas imaging typically does not reveal neurological abnormalities. Currently, HM does not have a proven effective treatment, but studies have shown that liver transplants can improve clinical symptoms [22]. Moreover, there are data suggesting that shunt embolization may reverse severe spastic paraplegia in patients with HM [23].

### 2.3. Portopulmonary Hypertension

Portopulmonary hypertension (POPH) is a serious pulmonary vascular disease associated with portal hypertension, which can occur with or without liver disease. The pathological changes observed in POPH are similar to those seen in other types of pulmonary hypertension (PH), characterized by endothelial dysfunction, pulmonary vasoconstriction, and vascular remodeling.

The proinflammatory state induced by the hepatic metabolic changes promotes pathological angiogenesis, leading to vascular remodeling through mechanisms such as leukocyte aggregation and protease production. Consequently, this process contributes to liver fibrosis as hepatic stellate cells are activated, causing endothelial injury and damaging vascular endothelial and smooth muscle cells, ultimately resulting in an oxidative stress state [24]. These alterations lead to a progressive increase in pulmonary vascular resistance and right ventricle overload, culminating in severe right heart failure. Several studies have shown that half of the patients with chronic liver disease (CLD) exhibit hyperdynamic circulatory syndrome, characterized by high cardiac output (CO) and low systemic vascular resistance. Furthermore, PH is exacerbated by increased pulmonary blood volume due to severe water and sodium retention. In advanced liver disease, there is an elevation in serum levels of vasoconstrictors (endothelin-1, thromboxane A2, norepinephrine and angiotensin II, and IL-1 and IL-6), along with a decrease in serum levels of vasodilators (prostaglandins and nitric oxide). These vasoactive substances contribute to pulmonary vasoconstriction and remodeling of endothelial and periarterial smooth muscle, thereby raising pulmonary artery pressure [25].

Not all patients with portal hypertension develop POPH, suggesting a potential role of genetic factors in its pathogenesis. Some studies have identified associations between high levels of estradiol and aromatase gene variants and an increased prevalence of POPH, suggesting the involvement of estrogen and its metabolites in POPH pathogenesis [26].

Risk factors for POPH include female sex, autoimmune hepatitis, SPSS, splenectomy, and anemia. While the severity of liver disease does not directly influence the incidence of POPH, the severity of cirrhosis can impact the survival of patients with POPH. DuBrock et al. investigated gender differences in the presentation and prognosis of POPH and found that females under the age of 50 had a lower survival rate, possibly due to the detrimental effects of estrogen and its metabolites on hemodynamic parameters and mortality risk in patients with POPH [27]. Determining the prevalence of POPH is challenging, but studies in the United States and Europe have reported a prevalence of 5% to 15% cases among cases of pulmonary arterial hypertension [28].

Patients with portal hypertension, especially those being considered for liver transplantation, should undergo evaluation for POPH using transthoracic echocardiography. Among patients awaiting liver transplants, echocardiography screening should be performed at least annually, although the optimal interval is yet to be determined.

For untreated patients, the prognosis of POPH is exceedingly poor, with only a 14% 5-year survival rate. Severe POPH is considered an absolute contraindication for liver transplantation (LT) [29]. Therefore, it is imperative to explore the risk factors for POPH and identify patients at high risk.

The diagnosis of POPH relies on clinical confirmation of portal hypertension and hemodynamic parameters obtained from right heart catheterization (RHC), which reveals a specific hemodynamic profile: mean pulmonary artery pressure (mPAP) > 20 mmHg, pulmonary vascular resistance (PVR) ≥ 3 Wood units, and pulmonary artery wedge pressure (PAWP) ≤ 15 mmHg, in the absence of other causes of PH, such as pulmonary disease, left heart disease, and connective tissue disease.

Although several factors have been identified to predict survival in patients with POPH, there are currently no specific indicators of treatment effectiveness. The primary treatment options include targeted pulmonary vasodilator therapy, liver transplantation once the pulmonary hemodynamic profile is optimized, and an exercise program. Specific medications for POPH aim to reduce pulmonary vascular resistance more than they decrease pulmonary artery pressure, as they also increase cardiac output, which may delay the anticipated improvement in mean pulmonary artery pressure. In patients with POPH, pulmonary arterial hypertension therapies have a unique role in facilitating liver transplants. Studies have shown that 44% of patients with POPH become eligible for liver transplant after receiving pulmonary arterial hypertension therapy [30]. Current therapies for pulmonary hypertension encompass several classes of medications, including prostacyclin agonists (e.g., epoprostenol), prostacyclin receptor agonists (e.g., selexipag), endothelin receptor agonists (e.g., bosentan), phosphodiesterase type 5 inhibitors (e.g., sildenafil), and guanylyl cyclase stimulants (e.g., riociguat). However, pulmonary arterial hypertension-specific medications often overlap with signs and symptoms of liver disease, such as nausea, anorexia, and edema, which may limit the aggressiveness of treatment [31]. Nevertheless, despite potential side effects, it is crucial to treat POPH, with the main goal of therapy being symptom relief and improvement in quality of life.

### 2.4. Portal Vein Thrombosis

Portal vein thrombosis (PVT) is increasingly recognized as liver disease progresses, particularly in the context of portal hypertension and portosystemic shunts. A recent retrospective study involving 222 cirrhotic patients found that SPSS were present in 63.5% of cases, with PVT and Child Pugh class C being independently associated with the presence of SPSS [32]. Other studies have reported a higher prevalence of PVT in patients with SPSS (30%) compared to those without SPSS (8%), with the incidence of PVT being closely linked to the size of the shunt. Moreover, the type of SPSS appears to influence the incidence of portal vein thrombosis, as patients with splenorenal shunts have a higher incidence compared to those with paraumbilical shunts [12]. The splenorenal shunt promotes the portal steal phenomenon, contributing to the development of portal vein thrombosis [33].

## 3. Imaging and Measurements

Over time, the methods used for diagnosing SPSS have evolved from invasive studies such as angiography, percutaneous transhepatic portography, or splenoportography, whose applicability was somewhat limited, to modern noninvasive imaging techniques represented by Doppler ultrasonography (US), computed tomography with contrast (CT), and magnetic resonance imaging (MRI).

Ultrasonography is the most accessible method of exploration when SPSS is suspected. It is an inexpensive method that allows for the evaluation of most shunts. In addition, ultrasonography enables the assessment of liver morphology, spleen size, and changes in the caliber of the portal vein and portal flow in patients with cirrhosis.

The limitations of ultrasonography depend upon the operator’s experience and the patient’s condition, such as obesity or acoustic interference caused by intestinal gas. Small SPSS and those deeply localized are often not identified. Even when visualized, a full assessment of the anatomy and caliber of collateralization pathways is difficult to perform [15].

The main role in the detection and assessment of SPSS belongs to the sectional methods represented by CT and MRI. Both imaging techniques can evaluate the entire abdomen, providing a comprehensive picture of the entire splenoportal system, and are less sensitive to variations in body volume or the presence of gas in the patient’s digestive tract.

CT examination appears to have advantages over other imaging methods due to its availability, rapid acquisition, high spatial resolution, and the capability for multiplanar or three-dimensional reconstruction [34]. MRI is equally accurate in assessing the anatomic course of collaterals, especially when using thin-section acquisitions and gadolinium-based contrast, with limitations related to availability, cost, and longer acquisition times.

SPSS can be classified based on their collateral drainage pathways. Some drain into the superior vena cava, with common examples including esophageal and paraoesophageal varices, gastric varices, and perisplenic varices. Others drain into the inferior vena cava, such as gastrorenal and splenorenal shunts, paraumbilical venous collaterals, and abdominal wall shunts.

**Figure 1:**
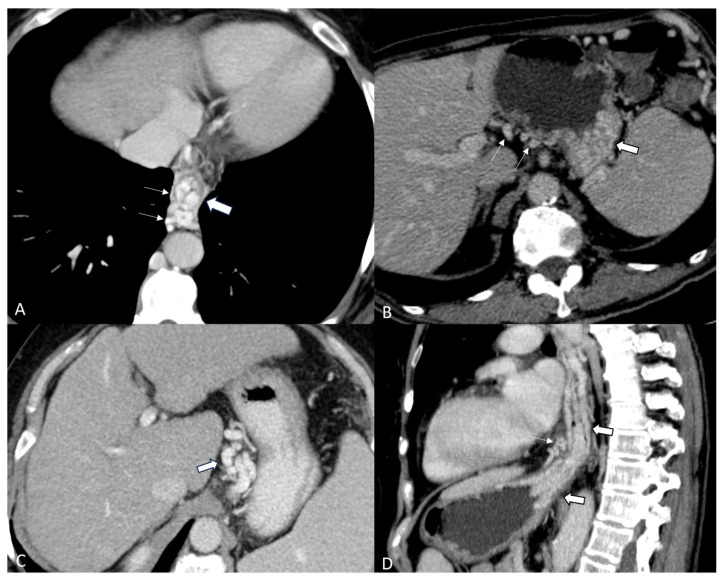
(**A**) CT appearances of esophageal varices show as intraluminal protrusion with scalloped margins (large arrow). Paraoesophageal varices (thin arrows) appear as well-defined, round, tubular, or serpentine structures situated outside the wall of the esophagus in the mediastinum. (**B**) Gastric varices (large arrow) located submucosal in the postero-superior aspect of gastric fundus and presence of dilated veins on the lesser curvature of stomach (thin arrows). (**C**) Left coronary vein varices (large arrow). (**D**) Gastroesophageal varices extend from inferior esophagus to the upper pole of the stomach (large arrows), in association with paraoesophageal varices (thin arrow).

Esophageal varices, representing the tortuous dilatation of the esophageal veins, are clearly visible on a CT examination in the venous phase as contrasting tubular structures adjacent to the esophageal mucosa or protruding into the lumen with round or scalloped edges. Paraoesophageal varices, frequently associated with esophageal varices, are venous dilatations that extend outside the esophageal wall and appear on post-contrast CT examinations as tortuous vessels extending along the distal esophagus (Figure 1A).

Gastric varices can be observed at the postero-superior portion of the gastric fundus (Figure 1B). They can develop in isolation, but most often, gastric and esophageal varices are present simultaneously and are considered a separate class called gastro-esophageal varices (Figure 1C).

Coronary varices occur when there is an increase in pressure within the portal system, leading to a reversal in the direction of blood flow. This results in hepatofugal blood escaping from the portal vein through the left gastric vein, causing the left gastric vein and its branches to become dilated and form a network of varicose veins. These varices are visible on post-contrast CT examinations, appearing as an enlarged and tortuous left gastric vein at the lesser curvature of the stomach and the posterior wall of the left hepatic lobe (see Figure 1D).

**Figure 2:**
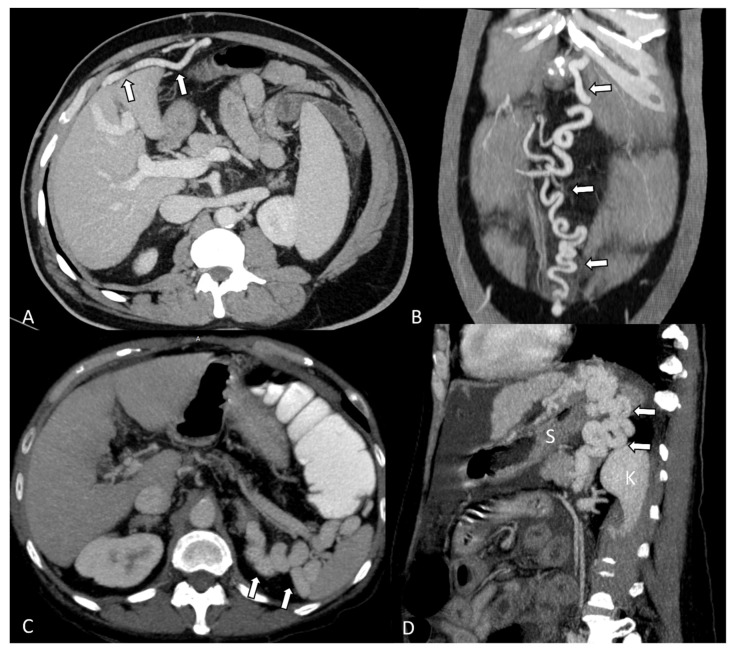
(**A**) Dilated paraumbilical veins anterior to the left hepatic lobe (arrows). (**B**) Abdominal wall varices radiating from the umbilicus, connecting cephalad with the internal mammary vein and caudally with the inferior epigastric vein (arrows). (**C**) Splenorenal shunt (arrows) appears as dilated and tortuous veins between the spleen and left kidney. (**D**) Gastrorenal shunt (arrows) between the coronary varices and the left renal vein, S—stomach, K—left kidney.

The paraumbilical veins, originating from branches of the left portal vein, become dilated in cases of portal hypertension and form anastomoses with the veins of the abdominal wall. On CT examinations, they are visible as tubular or round structures, following a descending trajectory along the round ligament to the umbilicus (Figure 2A).

Abdominal wall varices refer to dilated veins in the anterior abdominal wall. In severe cases of portal hypertension, the dilated veins can resemble a “caput medusa”, with tortuous veins radiating from the umbilicus (Figure 2B).

Spontaneous splenorenal and gastrorenal shunts form connections between the left renal vein and the splenic vein or its collaterals and between the left renal vein and either the coronary varices or the short gastric veins, respectively (Figure 2C,D).

Mesenteric collaterals can be subdivided into superior and inferior collaterals. Superior mesenteric collaterals originate from tributaries of the superior mesenteric vein: small intestine veins, cecal veins, colic veins (excluding the left colic vein), pancreato-duodenal veins, and right gastroepiploic veins. Inferior mesenteric collaterals originate from tributaries of the inferior mesenteric vein: superior rectal veins, rectosigmoid veins, sigmoid veins, and left colic vein.

Another advantage of CT examinations in evaluating spontaneous collateral pathways is their potential for noninvasive risk stratification of portosystemic shunts, with high sensitivity and specificity in patients with cirrhosis [35]. As liver disease progresses to advanced stages, the prevalence of spontaneous portosystemic shunts increases, often with more than one [14].

CT examinations provide an excellent spatial resolution, enabling the measurement of vessel diameters and identification of the path with the largest caliber. SPSS are classified according to the maximum diameter of the vessels into small (maximum diameter ≤ 8 mm) and large (maximum diameter of the shunt ≥ 8 mm) [36]. Patients with SPSS of an increased caliber (larger than 8 mm) are at a higher risk of decompensation [6].

In addition to these measurements, it is possible to calculate the total cross-sectional area of the shunts (TSA), which has a greater predictive value in the progression of cirrhosis. According to one study, a total cross-sectional area larger than 83 mm^2^ increases the risk of hepatic encephalopathy and mortality in patients with cirrhosis [14].

## 4. Medical Treatment

The association between the size of SPSS and liver function deterioration suggests that etiological treatment holds promise in halting the progression of both liver disease and SPSS. This notion was underscored in a study designed to evaluate the efficacy of etiological interventions, such as HCV treatment and alcohol abstinence, in arresting the advancement of SPSS. By assessing the SPSS distribution and liver function at baseline and during follow-up, the study revealed that patients with a worsening MELD score compared to their initial score exhibited an expansion of SPSS distribution [17]. Thus, despite the availability of medical resources, it is essential to prioritize the control of liver disease progression through etiological intervention whenever possible. At the same time, given the serious complications associated with SPSS, medical management involves treating HE, variceal bleeding, and porto-pulmonary hypertension.

## 5. Radiological Shunt Occlusion

Interventional radiology plays a pivotal role in addressing SPSS, offering a variety of practical approaches. These minimally invasive procedures provide significant advantages, including a reduced recovery time and fewer potential complications, making them a valuable option in the therapeutic arsenal for managing liver cirrhosis and its associated complications. Contemporary strategies for addressing SPSS involve several techniques.

Balloon-occluded retrograde transvenous obliteration (BRTO) is a method used to treat refractory HE by obstructing major types of spontaneous portosystemic shunts, specifically splenorenal and gastrorenal shunts. It is an efficient technique with a very low rate of recurrence of shunts [37]. The most common shunt occluded by the BRTO procedure is the gastrorenal shunt. This technique involves the placement of a balloon catheter within the gastrorenal shunt, selecting a balloon size that corresponds to the shunt’s diameter at its junction with the renal vein, effectively obstructing blood circulation. Subsequently, a sclerosing agent is administered directly into the gastric varices, inducing thrombus formation [38].

Plug-assisted retrograde transvenous obliteration (PARTO) is an alternative method proposed that replaces the balloon occlusion catheter and sclerosants with a vascular plug/coil and a gelatin sponge. The advantage of this method is to minimize some of the complications associated with balloon catheters and sclerosants [39].

Coil-assisted retrograde transvenous obliteration (CARTO) represents an adaptation of the BRTO procedure, employing coils as a substitute for the traditionally used indwelling balloon. This modification proves beneficial, particularly in scenarios where the anatomy of the shunt renders balloon placement impractical. In performing CARTO, the reported technique involves utilizing two microcatheter systems, designated separately for the deployment of coils and the injection of gel foam, with both catheters accessed through a dual-route approach [40].

Although infrequent, the primary complications linked to shunt embolization include hematuria, pulmonary edema, and shock. Additional potential adverse reactions encompass allergic responses to the sclerosing agent and portal thrombosis, which may occur if the sclerosant flows from the left gastric vein into the portal vein or from the splenorenal shunt into the splenic vein [38].

Closing large shunts can lead to a significant improvement in shunt-related hepatic encephalopathy due to the reduced shunt blood flow and decreased accumulation of toxins [8]. However, as liver disease progresses, multiple portosystemic shunts may develop, and closing a single shunt may not be sufficient to manage hepatic encephalopathy symptoms effectively. In cases where multiple shunts exist and mild hepatic encephalopathy remains after closing the gastrorenal shunt, it may be necessary to evaluate and consider closing the other shunts.

## 6. Surgical Approach

Another option for treating the consequences of SPSS is the surgical ligation of these normal connections developed between the portal venous system and the caval venous system. While there are no definitive studies on the surgical interruption of SPSS, the use of non-absorbable polymer clips such as Hem-o-lok, polypropylene sutures, or vascular sealing systems is suggested [41]. Surgical procedures are considered when endovascular radiological or endoscopic procedures fail, particularly when the veins are too tortuous to be catheterized or the shunt diameter is too large to be embolized. Theoretically, surgical intervention can also be considered when another surgical procedure, such as splenectomy or cholecystectomy, is necessary [42]. Although the indications for surgical intervention are like those for radiological embolization, they must be carefully weighed against the risks associated with general anesthesia, perioperative incidents, and complications of laparotomy or laparoscopy. The decision to opt for the surgical management of portosystemic shunts should be based on the patient’s overall condition, shunt flow, and major associated complications such as HE and esophagogastric variceal rupture. In the trend toward personalizing the optimal treatment for improving the quality of life of patients, surgical procedures remain a rarely used option.

## 7. Impact of Liver Transplantation

Liver transplantation (LT) has been a valuable therapeutic option for patients with end-stage liver disease [43]. After LT, most SPSS regress and become clinically insignificant. However, in 30–40% of cases, they may persist, causing reduced graft perfusion. The portal steal phenomenon is suspected when the portal vein velocity is lower than 20 cm/s, and a decrease below 10 cm/s requires immediate intervention [44].

The management of SPSS during liver transplantation (LT) is extensively debated. When left in place at LT, large SPSS (greater than 1 cm) have been associated with an increased risk of primary nonfunction (PNF), primary dysfunction (PDF), portal vein thrombosis (PVT), or HE due to decreased portal inflow to the graft secondary to the portal flow steal [33,45]. Ligation of SPSS can be technically challenging, carrying a high risk of bleeding in the context of severe portal hypertension, and may lead to inferior vena cava (IVC) thrombosis [46,47]. Some studies support the utility of preoperative percutaneous endovascular embolization if Doppler ultrasound shows slow hepatofugal flow in the portal vein. Other authors recommend that the decision to ligate SPSS should be based on an intraoperative assessment of inadequate flow to the allograft [4]. Small SPSS may have a protective role on the liver graft, restraining the portal hypertransfusion, transient portal hypertension, and sinusoidal damage, which are the main causes of small-for-size syndrome (SFSS) [48].

A prospective study analyzed the outcomes of SPSS ligation versus no ligation in 66 patients with SPSS undergoing LT. In 54.4% of cases, SPSS was ligated after portoportal anastomosis, while in 45.5% of cases, it was left in place. There was no significant difference in rates of PNF and PDF between the two groups. However, nonligation of SPSS was associated with a significantly higher rate of post-LT HE and major morbidity. Patient and graft survival were better in the group with ligated SPSS, supporting the benefits of routine SPSS ligation during LT whenever possible [2]. Similar results have been obtained by Gómez-Gavara et al. [49] in a study of 66 patients with SPSS > 1 cm undergoing LT. In approximately half of cases, shunt ligation was performed. The authors concluded that SPSS ligation was associated with lower postoperative morbidity, HE, and PVT and with better outcomes regarding patient and graft long-term survival over a follow-up period of 25 months. Recently, a novel tool was proposed for measuring the total shunt area (TSA), which allows for an evaluation of SPSS extension. Higher TSA correlates with lower patient survival. In a retrospective evaluation of 346 cirrhotic patients undergoing liver transplantation between 2015 and 2020, a TSA greater than 8.54 mm^2^ was found to be a risk factor for the development of early allograft dysfunction (EAD) and grade 3 acute kidney injury [50].

On the other hand, some studies have found no difference in clinical outcomes for patients with SPSS after LT, suggesting that it may not be necessary to correct SPSS intraoperatively [51]. A meta-analysis of 13 studies evaluated 962 patients with SPSS; during LT, 372 shunts (38.7%) were treated, and 590 (61.3%) were observed. After a follow-up period ranging from 4 months to 5 years, there were no differences in overall survival at 1 year between the two groups. Portal vein complications, such as stenosis, reduced flow, or thrombosis, were more likely in the SPSS-ligated group (2%) compared to the observed group (4%, *p* = 0.22). Thus, in this study, SPSS management during LT did not provide any advantages over non-intervention [52].

The management of SPSS in LT remains questionable. Current recommendations suggest considering SPSS ligation in patients at high risk for developing graft hypoperfusion, portal complications, or HE (e.g., very large shunts or low portal vein flow). SPSS ligation may be avoided in cases with small-sized grafts or when it is technically challenging. Shunt embolization might be considered if symptomatic large SPSS remains during long-term follow-up [51].

## 8. Conclusions

The development of SPSS in liver cirrhosis is a compensatory response to portal hypertension. The anatomical location of these shunts significantly affects associated clinical risks and complications. Contrary to initial beliefs, SPSS may increase the risk of variceal bleeding. Moreover, there is a well-documented link between SPSS and HE, with larger shunts contributing to a higher incidence of HE. SPSS can predispose patients to HE without additional precipitating factors, highlighting the need for thorough evaluation and management, especially in liver transplantation candidates.

Advanced imaging techniques like CT and MRI are crucial for detecting and assessing SPSS. CT scans provide precise measurements of vessel diameters and the largest pathways, with the total cross-sectional area offering predictive value for cirrhosis progression. Areas larger than 83 mm^2^ are associated with higher risks of HE and mortality. Therefore, early CT imaging is valuable for assessing portal hypertension and SPSS, offering essential data to guide subsequent monitoring and management.

The link between SPSS size and liver function deterioration indicates that treating the underlying cause of liver disease can help prevent both conditions from worsening. Therefore, it is crucial to prioritize etiological treatments to control liver disease progression. Additionally, managing SPSS-related complications like hepatic encephalopathy, variceal bleeding, and porto-pulmonary hypertension remains essential. Interventional radiology offers effective, minimally invasive solutions for SPSS-related complications, including BRTO, PARTO, and CARTO, which target major SPSS types like splenorenal and gastrorenal shunts. Surgical interventions are rare and used when less invasive methods fail.

Liver transplantation is a key treatment for end-stage liver disease, often resulting in the regression of SPSS. Intraoperative ligation of SPSS may be considered for patients at high risk of graft hypoperfusion, portal complications, or HE.

The multifaceted implications of SPSS require a multidisciplinary approach to optimize care for liver cirrhosis patients affected by these shunts.
